# Eagle's Syndrome Presenting With Peripheral Facial Palsy—A Case Report

**DOI:** 10.1002/hed.70305

**Published:** 2026-06-01

**Authors:** Vincent Holtmann, Roman Ackermann, Klaus Jahn, John‐Martin Hempel

**Affiliations:** ^1^ Department of Otolarnygology LMU Hospital Munich Bavaria Germany; ^2^ Department of Neurology Schön Klinik – Bad Aibling Harthausen Bad Aibling Bavaria Germany

**Keywords:** Eagle's syndrome, peripheral facial palsy, styloid process

## Abstract

**Background:**

Eagle's syndrome is a condition caused by an elongated styloid process and is characterized by various symptoms such as cervicofacial pain, foreign body sensation, headache, odynophagia, and otalgia. However, the occurrence of a unilateral peripheral facial palsy as a symptom of Eagle's syndrome has been described only rarely.

**Methods:**

We report the case of a 60‐year‐old male patient who presented with a sudden and unprovoked onset of retroauricular pain on the left side, accompanied by peripheral left facial palsy. Imaging revealed an extensively elongated styloid process on the left side. Based on the clinical and radiological findings, a spontaneous fracture of the styloid process with subsequent inflammatory reaction compromising the nearby facial nerve was suspected. Surgical resection of the elongated styloid process was performed.

**Results:**

Three months after surgery, the peripheral left facial palsy, which had shown no preoperative improvement for many weeks, had improved from House–Brackmann grade IV° to II°. No other symptoms were present postoperatively.

**Conclusions:**

We present a rare case of Eagle's syndrome complicated by unilateral peripheral facial palsy that improved following surgical resection of the elongated styloid process.

## 1 Introduction

Eagle's syndrome was first described in 1937 by W. Eagle and is caused by the elongation of the styloid process and/or the calcification of the styloid ligament, which attaches to the styloid process and extends to the small horn of the hyoid bone [1, 2]. Nevertheless, already in 1652 P. Marchetti was the first to report a case of an elongated ossified styloid process and J. Weinlechner was the first to describe a surgically treated case of an elongated styloid process for symptomatic relief in 1872 [3]. The elongation of the styloid process can occur unilaterally and bilaterally. The typical length of the styloid process falls within 2–3 cm, being considered as elongated when exceeding the 3‐cm mark [4]. However, the elongated styloid process can lead to various symptoms such as cervicofacial pain, foreign body sensation (e.g., globus sensation), headache, odynophagia, dysphagia, and otalgia and is then referred to as Eagle's syndrome [5, 6]. The data on the prevalence of an elongated styloid process are controversial, but Eagle described it with about 4% of the general population with most of them being bilateral and asymptomatic [7]. Only a small percentage, estimated between 4% and 10.3%, within this category, is expected to actually display symptoms [4].

Yet the occurrence of an unilateral peripheral facial palsy as a symptom of Eagle's syndrome has only been described very rarely. Generally, the facial palsy can be divided into two different types depending on the location of the lesion. A central facial palsy results from a supranuclear damage and a peripheral palsy from an infranuclear damage. The most common reason for peripheral facial palsy is Bell's palsy also referred to as idiopathic facial palsy, which has an uncertain cause though the activation of the herpes simplex virus is considered probable. Other factors leading to a peripheral facial palsy encompass trauma, particularly fractures of the petrous bone, iatrogenic damage, infectious diseases (e.g., Lyme disease, Ramsay Hunt syndrome, etc.) and compression or malignant infiltration of the facial nerve along its peripheral routing (e.g., parotid tumors, acoustic neuroma, cholesteatoma, etc.) [8].

Six cases regarding the occurrence of unilateral peripheral facial palsy as a symptom of Eagle's syndrome can be found in the literature [6, 8, 9, 10, 11, 12]. Five of them described a surgical resection of the elongated styloid process. To date, only a single case has been reported in which unilateral peripheral facial palsy associated with Eagle syndrome showed postoperative improvement following resection of the styloid process. Thus, we present a rare additional case of Eagle syndrome associated with unilateral peripheral facial palsy, in which facial nerve function improved following the surgical resection of the elongated styloid process.

## 2 Clinical Case

A 60‐year‐old male patient presented with a sudden and unprovoked onset of pain in the region of his left jaw angle. Initially, there were mainly chewing and swallowing pains which then slowly subsided. Additionally, 4 days after the first pain event, a peripheral left facial palsy graded as House–Brackmann II—III° developed. The next day, an oral cortisone therapy was initiated with initially 100 mg prednisolone for a total of 10 days, with the dose being reduced to 40 mg for the last 3 days. Infectious causes were excluded by laboratory tests including lumbar puncture and CSF analysis. Two weeks after starting the oral cortisone therapy, the patient presented again and showed a worsening of the left facial palsy to House–Brackmann IV°. In Figures 1 and 2 the amplitude loss of the left facial nerve from 2.4 to 0.3 mV after the oral cortisone therapy can be observed in the electroneuronography (ENOG). An MRI scan showed asymmetric distension of the styloid process on the left side with edematous infiltration of the surrounding tissue extending anteriorly into the *pterygopalatine fossa* (Figure 3). Although the predominant reactive changes are observed in the surrounding tissues anterior to the styloid process, it can be assumed that tissue lateral and posterior to the styloid process, extending toward the facial nerve, are also affected. In the deformed styloid process itself there were no suspicious signal changes in the sense of a tumorous process. Additionally, a CT scan showed a massively enlarged left styloid process with a max. diameter of 20.8 mm (Figures 4 and 5). The patient was then prepared for surgical treatment, which took place 6 weeks later, and prednisolone was again prescribed in the dose of 60 mg for 2 days, then 40 mg for 7 days, then 20 mg for 7 days, then 10 mg for 7 days.

**FIGURE 1:**
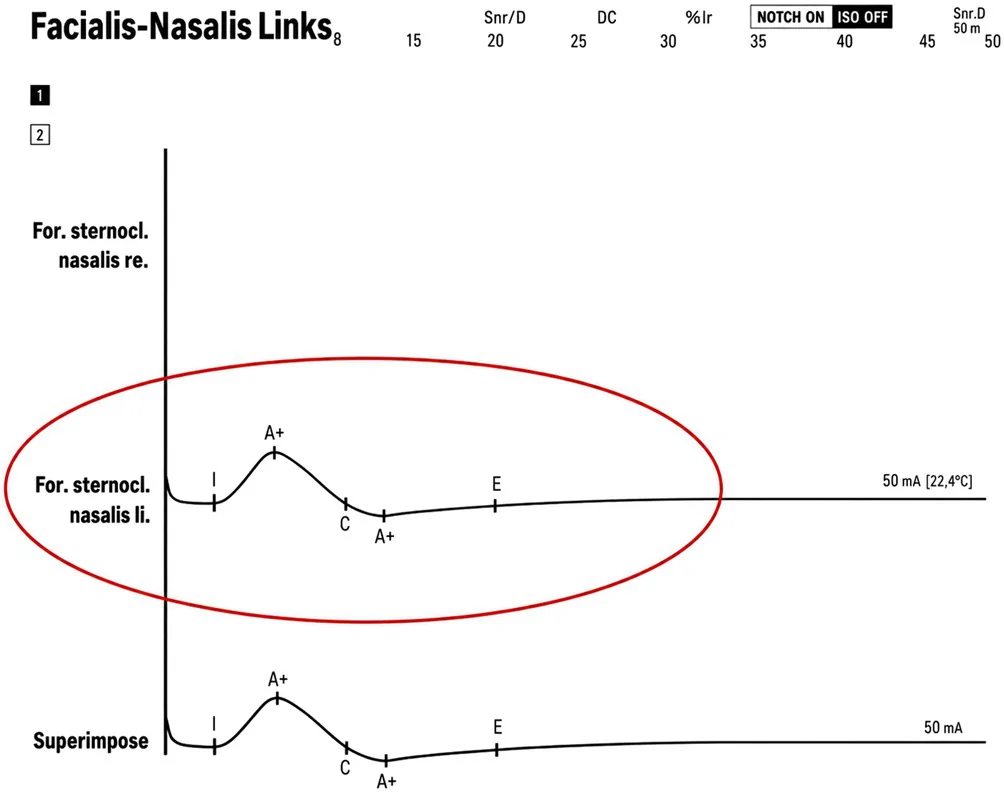
ENOG left facial nerve at the start of oral cortisone therapy.

**FIGURE 2:**
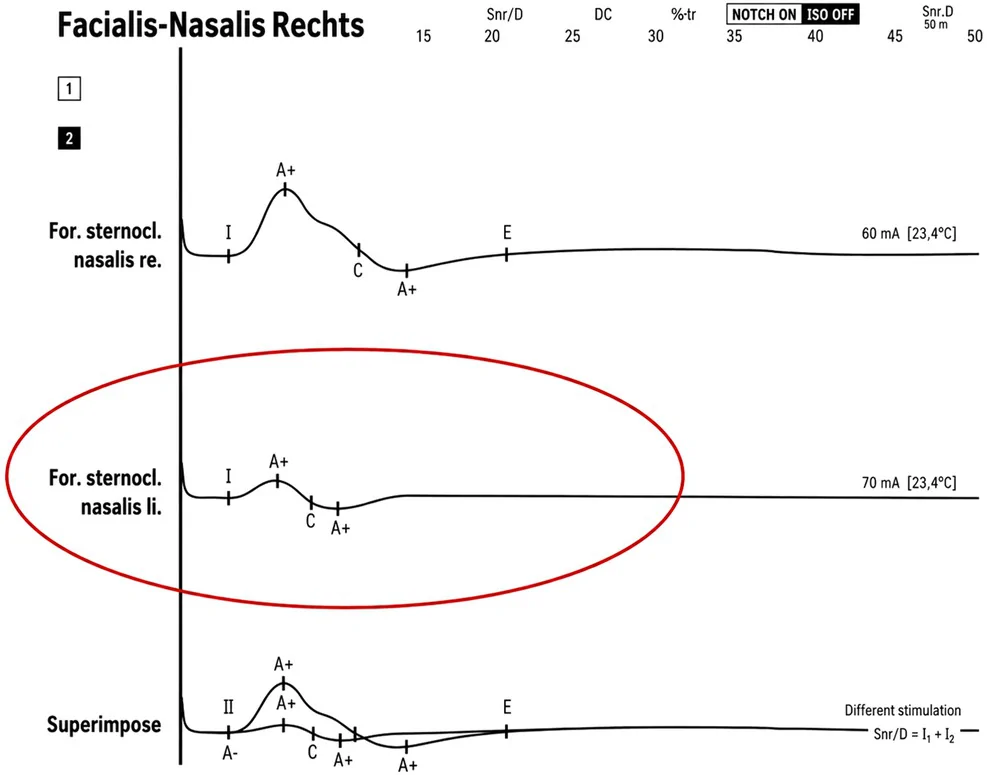
ENOG left facial nerve 2 weeks after initial oral cortisone therapy.

**FIGURE 3:**
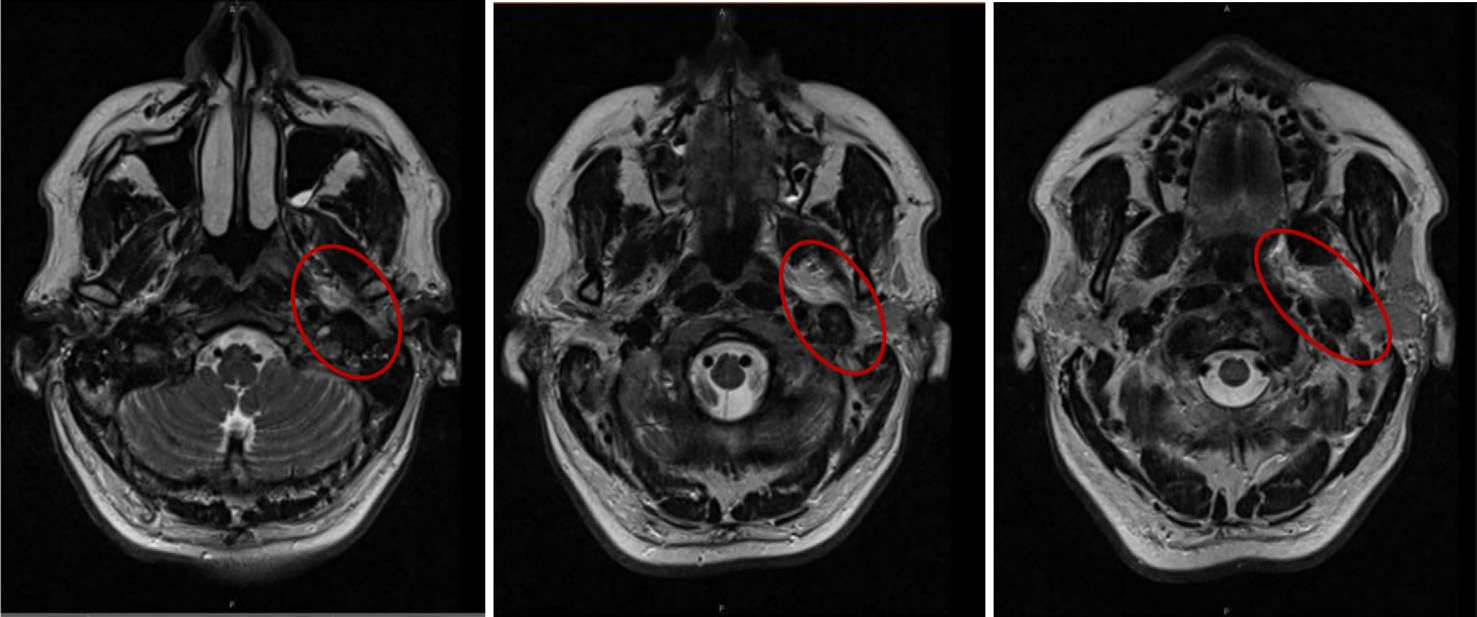
MRI scan (T2 TSE sequence—axial plane)—left styloid process with edematous reaction.

**FIGURE 4:**
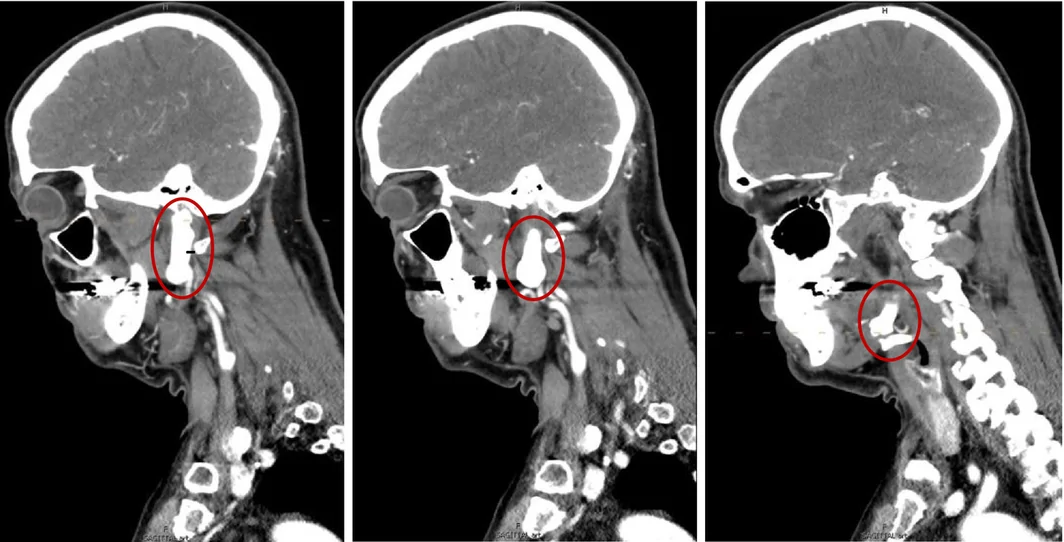
CT scan—elongation of the left styloid process (sagittal plane).

**FIGURE 5:**
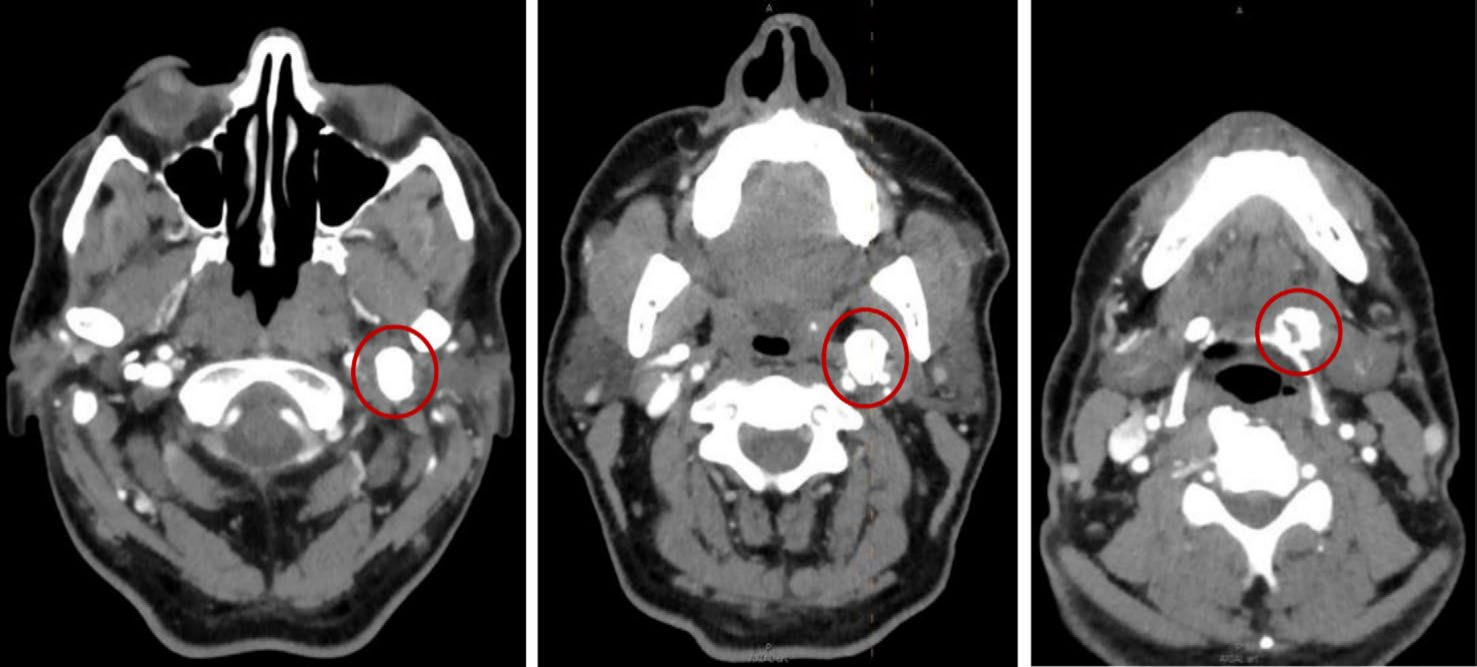
CT scan—elongation of the left styloid process (axial plane).

Two months after the first pain event the decision was made to undergo surgical treatment initially performing a microvascular decompression of the facial nerve using a retroauricular approach and C‐shaped skin incision. Due to the inflammatory changes in the tissue and the fact that the facial nerve did not respond to neuromonitoring, the attempt to visualize the facial nerve at the stylomastoid foramen was unsuccessful at first. Accordingly, the subtotal mastoidectomy was then carried out, exposing the usual anatomic landmarks. The facial nerve was then visualized distal to the stylomastoid foramen and accordingly the mastoid tip was ground down and the facial nerve was decompressed approximately 1.5 cm distal to the stylomastoid foramen. After obtaining tissue for the histological examination in the area of the stylomastoid foramen (Figure 6), which was affected by inflammation, the styloid process was visualized, and crepitus was observed. As a further step, the decision was made to remove the styloid process in the event that the histological results could rule out a malignant process.

**FIGURE 6:**
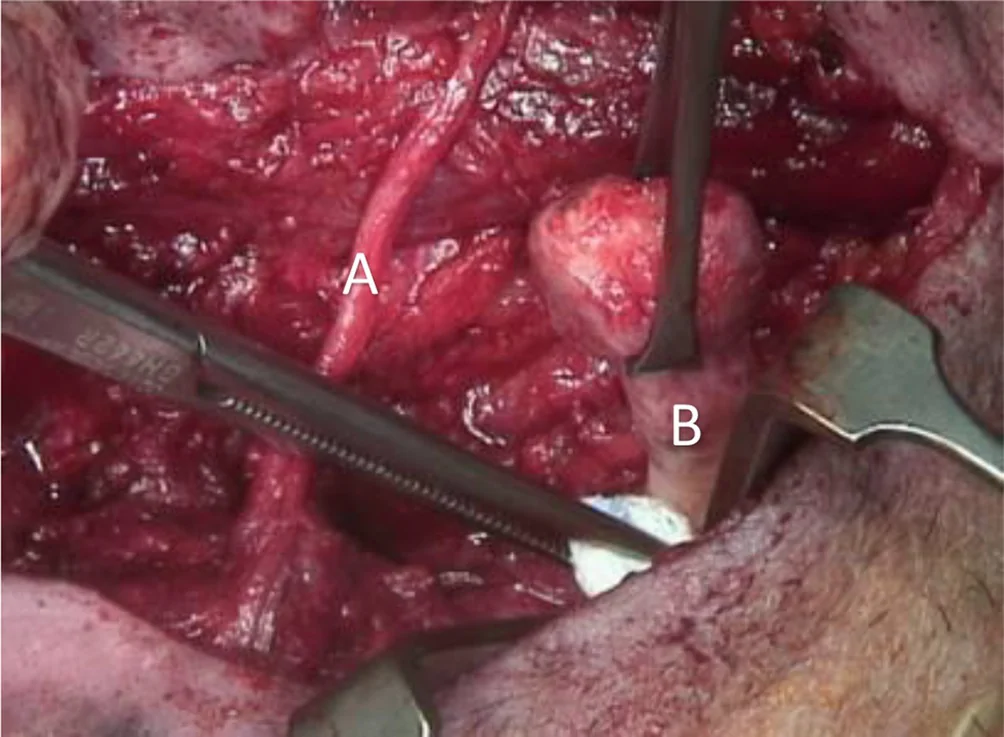
Extraction of the styloid process. (A) Great auricular nerve. (B) Styloid process.

Accordingly, after ruling out a malignant process in the histological examination, the surgical resection of the styloid process was attempted within the next week using the same approach. For this purpose, the styloid process had to be freed from the surrounding tissue with great effort due to the impression that inflammation with adhesion of the styloid process and ossified stylohyoid ligament with the surrounding tissue must have taken place. In order to be able to carry out the resection, the styloid process had to be split. With again great effort, the ossified ligament was separated from the hyoid bone and, while protecting all relevant anatomic landmarks, the styloid process was removed (Figures 6 and 7).

**FIGURE 7:**
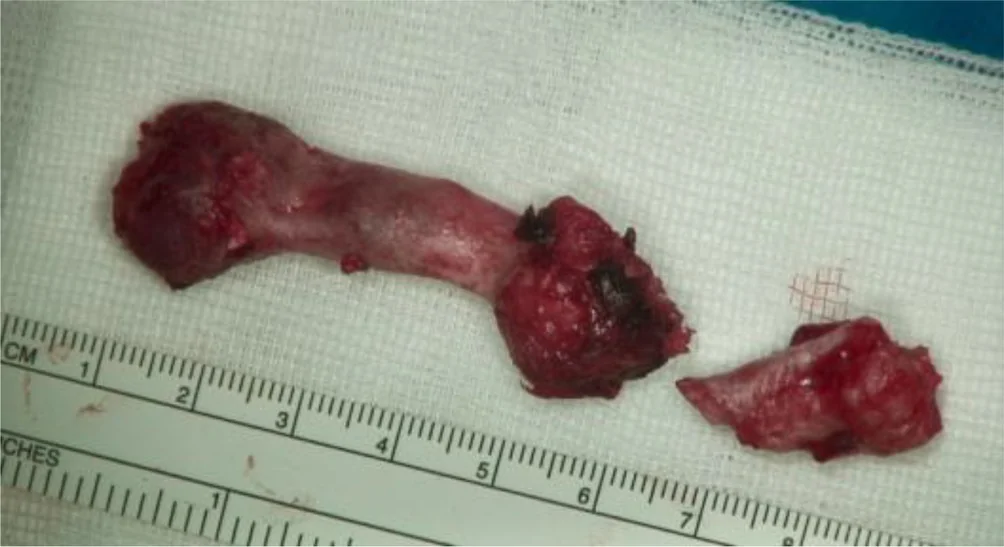
Elongated styloid process and ossified stylohyoid lig. (split).

After a histopathological examination, ossified structures were confirmed: The specimen consisted of lamellar, partly sclerotic corticospongious bone with pronounced remodeling activity and areas of endochondral ossification accompanied by prominent osteoblastic rimming. In addition, ligamentous soft tissue components and unremarkable hematopoietic islands were present, with no evidence of cellular atypia or malignancy. Overall, the findings were consistent with the clinical impression of an ossified ligament.

Almost 3 months after the surgical resection of the styloid process the patient described no symptomatic complaints and the facial palsy had reduced from House–Brackmann grade IV° to grade II°. In Figure 8, the patient is shown after the 2‐week oral cortisone therapy and thus still preoperatively (HB IV°). Here, the involvement of the temporal branches of the left facial nerve can be observed, indicating a reduced innervation of the left forehead, which is a clinical sign of a peripheral facial palsy. In Figure 9, the patient is shown postoperatively and after convalescence (HB II°) in comparable positions.

**FIGURE 8:**
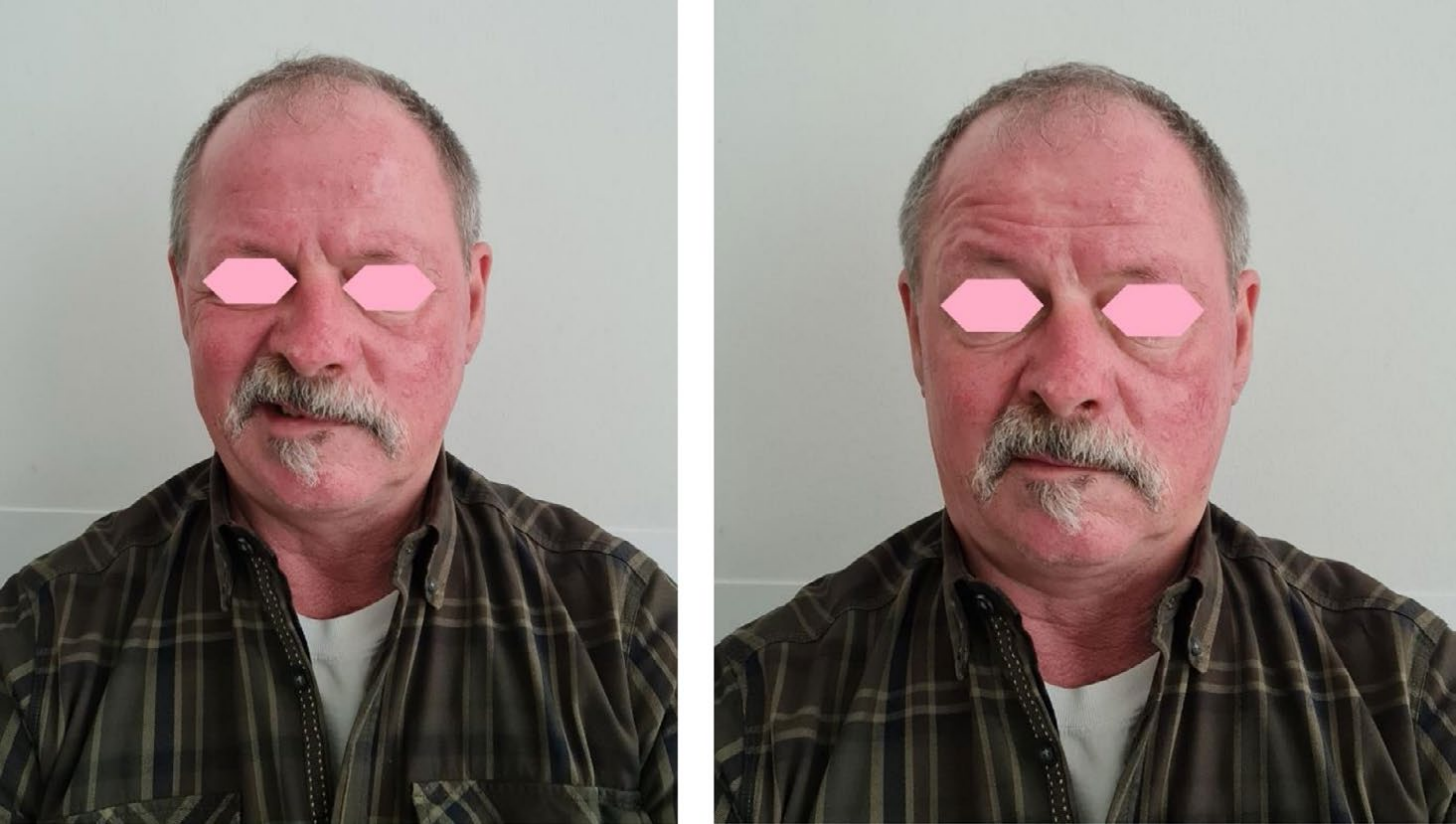
Preoperatively (HB IV°).

**FIGURE 9:**
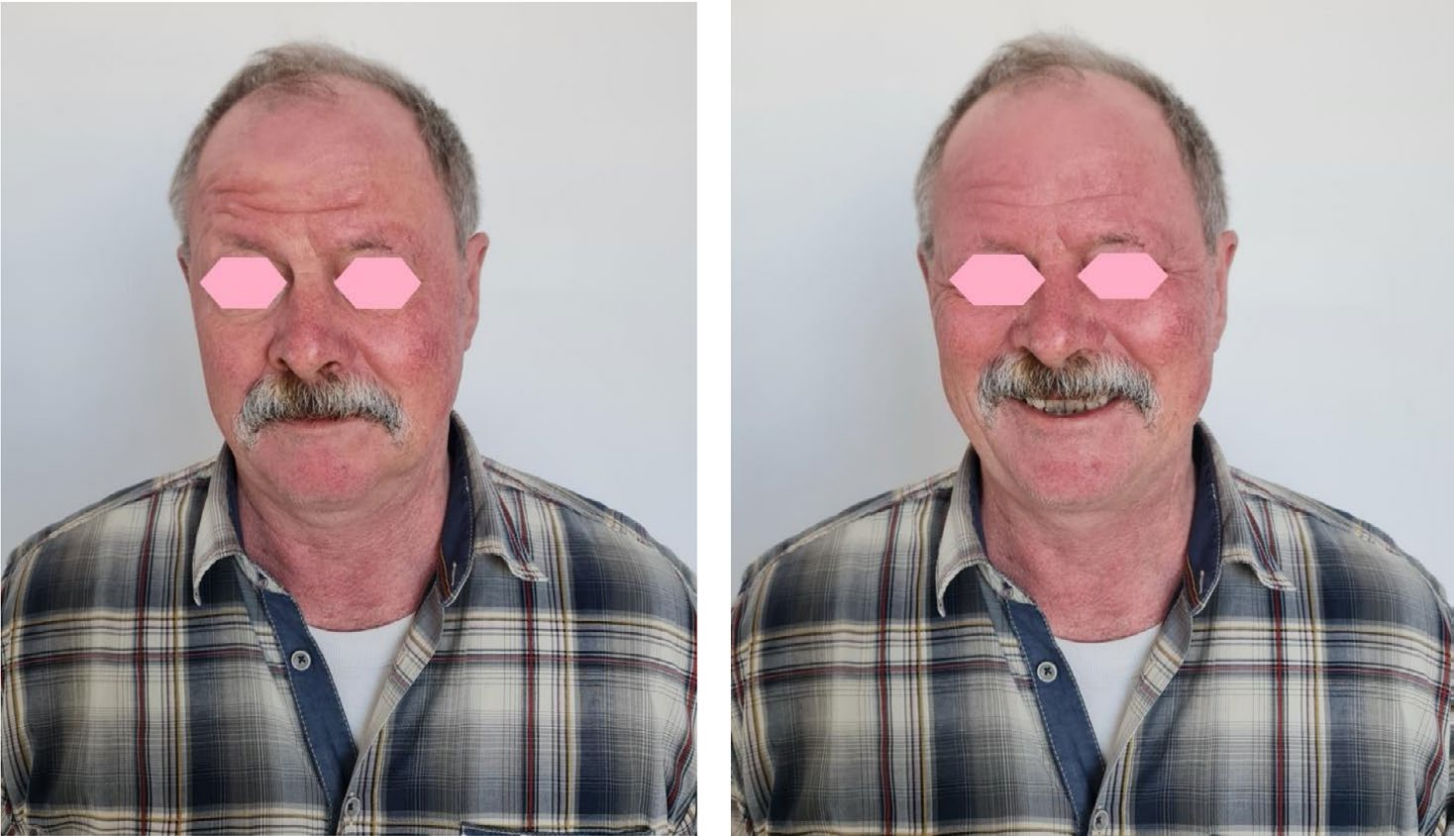
Postoperatively and after convalescence (HB II°).

## 3 Discussion

In this case report a patient has been presented who not only reported a sudden onset of pain on the left side, but also displayed a progressive unilateral peripheral facial palsy since then on the left side. In addition, the radiological correlate was a significantly elongated and thickened styloid process, which far exceeded 3 cm in length. This circumstance resulted in the diagnosis of Eagle's syndrome, although the occurrence of an unilateral peripheral facial palsy in this context is considered a rare phenomenon. Depending on the extent of the clinical symptoms, Eagle's syndrome can be treated both conservatively and surgically. Conservative therapy primarily includes the systemic administration of nonsteroidal anti‐inflammatory drugs or local, long‐acting anesthetic infiltration at the tonsillar fossa. The surgical therapy includes two approaches, depending on the anatomical location of the styloid process. Either through a transoral access if the styloid process is situated in the tonsillar fossa or parapharyngeal space or through an open access performing a cervical incision [8]. In this case, an open approach was chosen due to the location and size of the styloid process and the clinical symptoms. Accordingly, the styloid process was resected under great effort due to its size and the inflammation of the surrounding tissue.

As already mentioned, the occurrence of unilateral peripheral facial palsy as a symptom of Eagle's syndrome has been described very rarely. In a few of these cases a traumatic fracture of the styloid process may have played an important role in the emergence of Eagle's syndrome and hence also the peripheral facial palsy [6, 8, 11]. Similarly, a working hypothesis in this case is that a suspected spontaneous fracture of the extensively elongated styloid process may have played the central role in the emergence of Eagle's syndrome and hence also the peripheral facial palsy. As a result of the subsequent inflammation of the surrounding tissue after the fracturing of the styloid process, the symptoms appear to aggravate, leading to damage to the nearby facial nerve and a subsequent facial palsy.

Another working hypothesis is presented by Hoffmann et al. in a recent case from 2025. In this case, unilateral facial palsy was attributed to a markedly elongated and hypertrophied styloid process in combination with pseudoarthrosis at its base, resulting in pathological mobility. This abnormal mobility led to dynamic, position‐dependent mechanical compression of the facial nerve at the stylomastoid foramen, clinically manifesting as facial spasms during head rotation. Additionally, bony overgrowth proximal to the pseudoarthrosis caused partial stenosis of the distal mastoid segment of the facial canal, contributing to progressive nerve entrapment. The interplay of dynamic compression and static canal narrowing was considered responsible for the progression from facial spasms to complete ipsilateral facial palsy. Mechanical compression by the mobile giant styloid process was regarded as the primary etiological factor [12]. Although no facial spasms during movement were observed in our case, this report nevertheless provides an additional important hypothesis regarding the possible cause of unilateral peripheral facial palsy in the presence of an elongated styloid process. Moreover, this is consistent with our case, in which the styloid process is markedly expanded in width, with a maximum diameter of 20.8 mm, due to bony overgrowth and thereby compressing the surrounding structures.

An unusual feature in our case is not only the elongation but also the width, with a maximum diameter of 20.8 mm, as the styloid process—when elongated—is typically rather thin. The exact reason for the widening of the styloid process in this case cannot be determined with certainty. However, it is conceivable that, for example, a fracture may have initiated osseous remodeling processes, resulting not only in an inflammatory reaction but also in an increase in bony width. Alternatively, the previously described possible pseudoarthrosis and the associated pathological mobility, which may cause mechanical irritation, could also lead to bone remodeling. In more detail regarding the elongation of the styloid process (Type I Eagle syndrome), the most likely consideration is true osseous hyperplasia with formation of cancellous bone and increased bone density [13]. Although, as mentioned, a styloid process is generally considered elongated when exceeding 3 cm, elongation is relatively common in the general population and frequently asymptomatic. But age‐related degenerative ossification processes have been implicated in progressive elongation, with significantly greater lengths reported in older individuals [14, 15, 16]. A second important differential is a stylohyoid ligament ossification (Type II Eagle syndrome), which represents ligamentous metaplasia and ossification rather than true bony elongation of the styloid process itself. In rare cases, complete ossification of the stylohyoid complex may occur, potentially extending to adjacent ligamentous structures [13].

In our case almost 3 months after the surgical removal of the styloid process the patient's facial palsy could be reduced by two out of six grades from House–Brackmann grade IV° to grade II°. This improvement also makes the causality between the occurrence of an enlarged styloid process in combination with a fracture‐related/pseudoarthrosis‐related inflammatory reaction and the development of a facial palsy likely. However, it is not clear whether an elongated styloid process is more prone to fractures than a normally configured styloid process and also which role fractures might play in predisposing to a reactive consecutive enlargement of the styloid process in the first place.

Another aspect which supports the hypothesis that in this case it is not an idiopathic peripheral facial palsy is the fact that the palsy did not improve despite oral cortisone therapy, but on the contrary, it worsened. This was evident not only clinically (Figure 9) but also in the ENOG (Figures 1 and 2).

Remarkable is also the fact that there was a clear improvement despite the relatively long period between the appearance of the peripheral facial palsy and the surgical resection. Due to the long duration of the severe peripheral neurogenic facial palsy, a rather poor prognosis would have been expected. Nevertheless, the surgical intervention proved to be an effective therapy, which must therefore be considered even after a prolonged existence of a peripheral facial palsy in this context.

In summary, eventually it remains to be clarified how high the risk of developing Eagle's syndrome and an unilateral peripheral facial palsy due to a fractured/pseudoarthrotic styloid process is. Since this is a relatively rare clinical picture, studies with larger numbers of cases to more precisely explore and determine the connection between Eagle's syndrome and unilateral peripheral facial palsy are unlikely. Accordingly, attention must be paid to this possible connection in future similar cases and patients would need to be followed up in the long term in order to further document the progression of the facial palsy and to be able to make a lasting assessment of whether the actual cause could be remedied.

In addition, it must be mentioned that an idiopathic peripheral facial palsy, which still represents the majority of peripheral facial palsies, cannot be ruled out but must be considered highly unlikely in this context regarding all the presented findings. Thus, this case shows that when it comes to an idiopathic unilateral facial palsy together with additional symptoms like pain and foreign body sensation, Eagle's syndrome and a potential surgical intervention should also be taken into consideration.

## Data Availability

The data that support the findings of this study are available from the corresponding author upon reasonable request.
